# Chronic disease management in patients with intellectual disabilities: a matched study in Dutch general practice

**DOI:** 10.3399/BJGP.2023.0029

**Published:** 2023-09-05

**Authors:** Milou van den Bemd, Maarten Cuypers, Bianca WM Schalk, Geraline L Leusink, Erik WMA Bischoff

**Affiliations:** Department of Primary and Community Care, Radboud University Medical Centre, Nijmegen, the Netherlands.; Department of Primary and Community Care, Radboud University Medical Centre, Nijmegen, the Netherlands.; Department of Primary and Community Care, Radboud University Medical Centre, Nijmegen, the Netherlands.; Department of Primary and Community Care, Radboud University Medical Centre, Nijmegen, the Netherlands.; Department of Primary and Community Care, Radboud University Medical Centre, Nijmegen, the Netherlands.

**Keywords:** chronic disease, delivery of health care, disease management, general practice, healthcare disparities, intellectual disability

## Abstract

**Background:**

Disease management programmes (DMPs) aim to deliver standardised, high- quality care to patients with chronic diseases. Although chronic diseases are common among people with intellectual disabilities (ID), this approach may be suboptimal for meeting their care needs.

**Aim:**

To examine differences between patients with and without ID who have a chronic illness in DMP enrolment and disease monitoring in Dutch general practice.

**Design and setting:**

Observational study utilising the Nivel Primary Care Database (2015–2018) comparing patients with ID and cardiovascular disease, diabetes mellitus, or chronic obstructive pulmonary disease (COPD) with matched (1:5) controls with these conditions but without ID.

**Method:**

Using conditional logistic regression, enrolment in DMP per chronic disease was examined and differences tested between groups in the frequencies of consultations, medication prescriptions, and routine examinations.

**Results:**

A total of 2653 patients with chronic illness with ID were matched with 13 265 controls without ID. Patients with both diabetes mellitus and ID were more likely than controls to be enrolled in DMP (odds ratio [OR] = 1.44, 95% confidence interval [CI] = 1.27 to 1.64). Independent of DMP enrolment, patients with chronic illness with ID were more likely than controls to have frequent consultations. Patients with both diabetes mellitus and ID and patients with both COPD and ID who were not enrolled in DMPs had more medication prescriptions than non-enrolled patients with diabetes or COPD but without ID (OR = 1.46, 95% CI = 1.10 to 1.95; OR = 1.28, 95% CI = 0.99 to 1.66, respectively). Most patients with ID and their controls enrolled in DMPs received routine examinations at similar frequencies.

**Conclusion:**

Although DMPs do not specifically address the needs of patients with both chronic illness and ID, these patients do not seem underserved in the management of chronic diseases in terms of consultation, medication, and tests.

## INTRODUCTION

To reduce the high impact of chronic diseases, disease management programmes (DMPs) have been introduced in primary care in countries such as Germany, Sweden, the UK, the US, and the Netherlands.^1^ DMPs are multidisciplinary efforts to improve the quality and efficiency of chronic disease management by providing continuous, patient-centred, comprehensive care.^2^^,^^3^ DMPs encompass the management of patients with high-impact chronic diseases, such as cardiovascular disease (CVD), diabetes mellitus, or chronic obstructive pulmonary disease (COPD).^4^ In the Netherlands, chronic disease care is regionally coordinated by general practice care groups that share responsibility for delivering DMPs to their patients. Quality of care is assured by developing DMPs in line with national disease monitoring standards and by benchmarking practices’ performance against national criteria,^4^ thereby potentially preventing deterioration and complications in chronic diseases while maintaining a high quality of life.^5^^,^^6^

For some patient groups, such as patients with intellectual disabilities (ID), reducing the impact of chronic diseases is more complex. Although around 1%–1.5% of people globally are diagnosed with ID, the actual prevalence is likely to be higher, as not all IDs are recognised in general practice.^7^ People with ID experience significant limitations in intellectual functioning and adaptive behaviour, often manifested as difficulties recognising disease symptoms and understanding disease consequences, and, consequently, have increased health needs and health problems compared with people without ID.^8^^–^^13^ People with ID therefore have to avail of care more than people without ID.^8^^,^^14^ Moreover, chronic diseases such as CVD, diabetes mellitus, and COPD are more prevalent among people with ID, develop at younger ages, and occur more often in males with ID than in females with ID.^15^ These differences are seldom addressed in existing literature, disease guidelines, or DMPs.^16^

Despite these distinct characteristics, chronic disease management for patients with ID is provided mostly within the non- ID-oriented setting of general practice, which can be challenging for GPs. GPs have expressed difficulties in providing care to patients with ID, mostly relating to communication, continuity of care, and time constraints.^17^ These difficulties are reflected in compliance with guidelines. Although previous studies do not take DMP enrolment into account, most show that routine examinations advocated in DMPs are provided less frequently for patients with than without ID.^18^^–^^24^ Non-compliance with protocols and guidelines not only compromises quality of care, but may also contribute to health disparities, and puts patients with both chronic illness and ID at increased risk of complications, avoidable hospital admissions, and even premature mortality.^25^^,^^26^

**Table:** How this fits in

Because of the different chronic disease patterns and care needs of people with intellectual disabilities (ID), providing such patients with suitable chronic disease care within non-ID-oriented disease management programmes (DMPs) in primary care may be challenging. Although enrolment in DMPs was similar for patients with chronic illness with and without ID, those with ID who were not enrolled received routine examinations less often and received medication prescriptions more often. Most patients with and without ID enrolled in DMPs received routine examinations at similar high frequencies. Although DMPs do not specifically address the needs of patients with both chronic illness and ID, these patients do not seem underserved in the management of chronic diseases in terms of consultation, medication, and tests.

To tackle these inequalities, it is essential to strengthen the evidence base for providing adequate, suitable care for patients with chronic illness and with ID. Although DMPs promise quality of care, the complexity of care for patients with ID highlights the need for insights into any differences from the general population in DMP enrolment and disease monitoring. This study therefore aimed to examine differences between patients who had both chronic illness and ID and their controls who had chronic illness but not ID in terms of enrolment in DMPs and disease monitoring in Dutch general practice.

## METHOD

### Study design and data sources

This observational matched study used the Nivel Primary Care Database (NPCD), which routinely collects information from the medical records of >1 million patients registered in a representative sample of Dutch general practices.^27^^,^^28^ Only patients with a CVD, diabetes mellitus, or COPD diagnosis in their 2018 medical record were selected, using International Classification of Primary Care (ICPC) version 2 codes (see Supplementary Table S1).^29^ To advance ID identification, population data from Statistics Netherlands were linked to NPCD, described in more detail elsewhere.^7^ This study followed the Strengthening the Reporting of Observational Studies in Epidemiology (STROBE) guidelines for observational studies.^30^

### Study groups

For patients with a CVD, diabetes mellitus, or COPD diagnosis, data were collected retrospectively from 2015 onwards, or from diagnosis onwards. Patients with ID were identified by the presence of ICPC code P85 ‘Mental retardation’ in their medical record or when ID indicators were found through data linkage with ID-related social support or long-term care.^7^ Selecting all adults (aged ≥18 years) with ID and a chronic disease, the authors matched them randomly to five people without ID indicators in the same 10-year age group, of the same sex, and with the same chronic disease.

### Operationalisations

Patients were coded as being enrolled in a DMP if they had a record of enrolment in a DMP, if their GP was being coded as the main provider for chronic illness, or when >75% of the indicators, as advised in the DMP care guidelines, were fulfilled (see Supplementary Figure S1).^31^ Patients with solely a CVD diagnosis could be admitted to the CVD-DMP, patients with diabetes mellitus (with or without CVD or COPD) to the diabetes mellitus-DMP, and patients with COPD (with or without CVD or diabetes mellitus) to the COPD-DMP.

To examine disease monitoring, first consultations and medication prescriptions were examined. The mean of all consultations between 2015 and 2018 for all patients with CVD, diabetes mellitus, and COPD were calculated separately (weighted by patients’ registration time in general practice). The average (weighted) number of consultations (9.1 consultations per year for CVD, 8.3 for diabetes mellitus, and 9.3 for COPD) was used as the cut-off point to distinguish between individuals who were less and more ‘frequent consulters’. Similar calculations were used for medications based on prescriptions for CVD, diabetes mellitus, or COPD. The average (weighted) number of prescriptions between 2015 and 2018 for all patients with CVD, diabetes mellitus, or COPD (1.8 prescriptions per year) was used as a cut-off point to distinguish between less and more frequent users.

Second, frequency of key routine examinations in 2018 were examined. Several indicators considered essential for the monitoring of chronic disease according to Dutch and international guidelines were found.^4^^,^^21^^,^^32^^,^^33^ For CVD, these were the presence of a record of low-density lipoprotein (LDL) measurement for those aged <80 years and of a blood pressure test. For diabetes mellitus, these were presence of a record of haemoglobin A1C (HbA1c) measurement and an albuminuria test. For COPD, these were records of smoking behaviour and having ≥2 prescriptions for inhalation medication (R03A or R03B).

### Statistical analysis

Descriptive statistics of the study groups were presented as frequencies with percentages or as means with standard deviations (SDs). Frequencies with percentages were presented for the proportion of patients enrolled in DMPs, consultations, medication prescriptions, and adherence to routine examinations. Information on the latter three was stratified by enrolment in DMPs.

Differences between patients with ID and controls were compared using conditional logistic regression, estimating odds ratios (ORs) and 95% confidence intervals (CIs). *P*-values <0.05 were considered statistically significant. All analyses were conducted in IBM SPSS Statistics (version 25.0).

## RESULTS

### Demographics

Patients with ID and at least one chronic illness (*n* = 2653) were identified and matched with 13 265 controls without ID (Table 1). Most patients with and without ID were male (57.3%) and aged between 51 and 70 years (58.5%). Most patients with ID had a diagnosis of diabetes mellitus (59.7%. *n* = 1584), followed by CVD (31.9%, *n* = 845), and COPD (27.7%, *n* = 736). Of patients with ID, 62.0% (*n* = 1646) were registered 3.1–4 years at the GP compared with 59.3% (*n* = 7861) of controls. Of patients with ID, 70.2% (*n* = 593) had an indication for CVD- DMP, compared with 69.8% (*n* = 2952) of controls.

**Table 1.** Descriptive statistics of patients with and without intellectual disabilities with chronic diseases

**Characteristic**	**Patients with ID, *n* (%)^a^**	**Control group, *n* (%)^a^**
**Total**	2653	13 265

**Sex**		
Males	1519 (57.3)	7595 (57.3)
Females	1134 (42.7)	5670 (42.7)

**Age, mean (SD)**	54.7 (13.7)	55.2 (13.9)

**Age, years**		
18–30	193 (7.3)	965 (7.3)
31–50	644 (24.3)	3220 (24.3)
51–70	1553 (58.5)	7765 (58.5)
≥71	263 (9.9)	1315 (9.9)

**Registration time at GP, years**		
≤1	370 (13.9)	1751 (13.2)
1.1–2	468 (17.6)	2834 (21.4)
2.1–3	169 (6.4)	819 (6.2)
3.1–4	1646 (62.0)	7861 (59.3)

**Diagnosis of chronic disease^b^**		
CVD	845 (31.9)	4228 (31.9)
Indication for CVD-DMP^c^	593 (70.2)	2952 (69.8)
Diabetes mellitus	1584 (59.7)	7575 (57.1)
Indication for diabetes mellitus-DMP^c^	1584 (100.0)	7575 (100.0)
COPD	736 (27.7)	3680 (27.7)
Indication for COPD-DMP^c^	736 (100.0)	3680 (100.0)

a *Unless otherwise stated.*

b *Prevalence of chronic diseases is estimated with diagnoses in medical records based on International Classification of Primary Care codes.*

c *If patients had diabetes mellitus as well as CVD, they were admitted to diabetes mellitus-DMP. All patients with a diagnosis of COPD had an indication for the COPD-DMP. COPD = chronic obstructive pulmonary disease. CVD = cardiovascular disease. DMP = disease management programmes. ID = intellectual disabilities. SD = standard deviation.*

### Enrolment in DMPs

**Figure 1.**
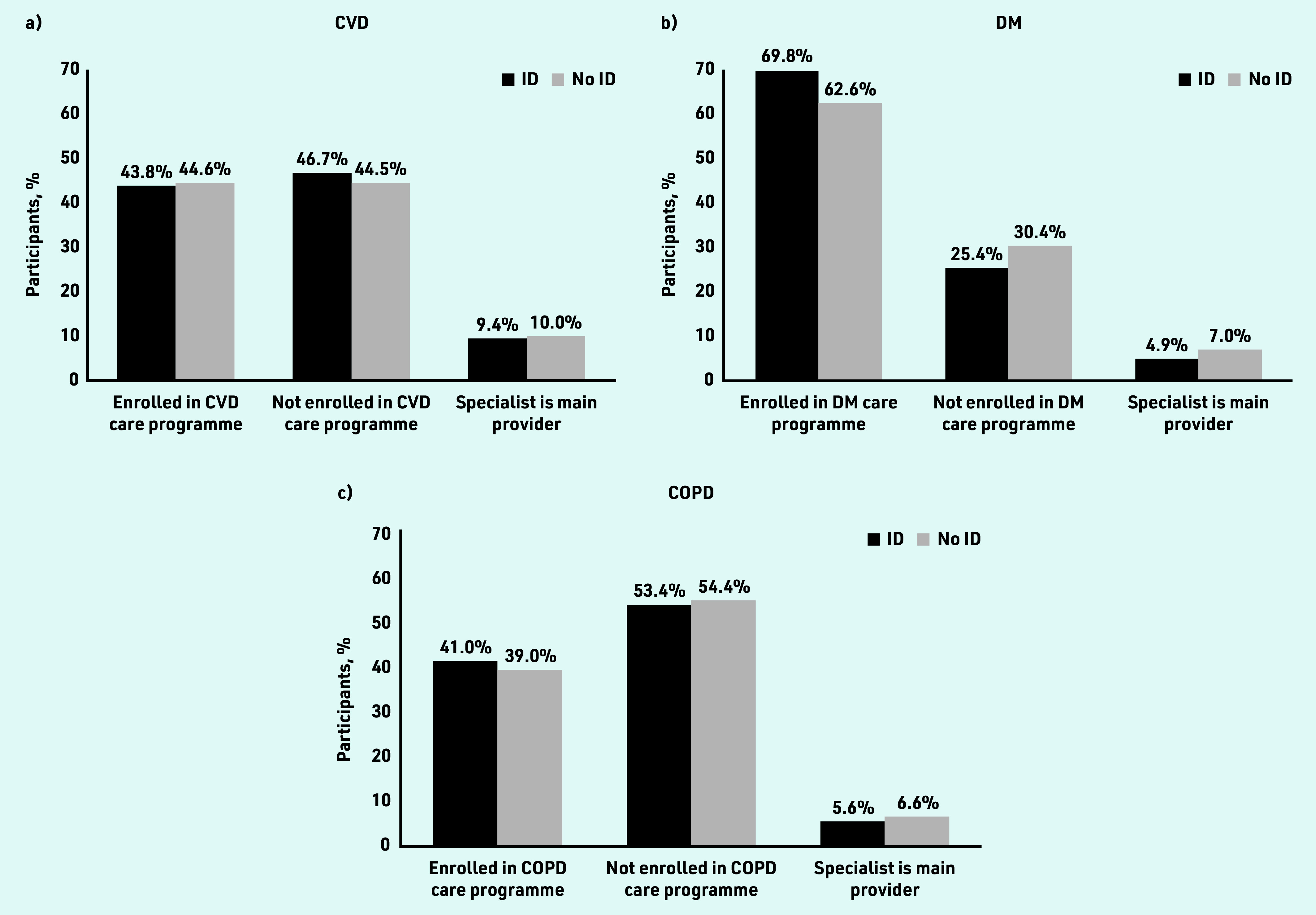
*Distribution of patients with and without ID by type of management for their chronic illness. a) CVD; b) diabetes mellitus; and c) COPD. COPD = chronic obstructive pulmonary disease. CVD = cardiovascular disease. DM = diabetes mellitus. ID = intellectual disabilities.*

Enrolment of people with ID in DMPs for CVD (43.8%) and COPD (41.0%) was comparable and not statistically significantly different from controls (Figure 1). Patients with diabetes mellitus and ID were more likely to be enrolled in a DMP than controls (69.8% versus 62.6%, OR 1.44, 95% CI = 1.27 to 1.64; Supplementary Table S2) and received diabetes mellitus care in specialist care settings less often than their controls (4.9% versus 7.0%, OR 0.65, 95% CI = 0.51 to 0.84).

### Disease monitoring

For those enrolled in DMPs, patients with chronic illness and ID were more likely than their controls to be frequent consulters: that is, for patients with ID and CVD (OR 2.71, 95% CI = 1.91 to 3.84), diabetes mellitus (OR 2.49, 95% CI = 2.14 to 2.89), and COPD (OR 3.01, 95% CI = 2.18 to 4.16; Table 2). Similar results were found for those not enrolled in DMPs.

**Table 2.** Frequency and percentage of patients with chronic illness with and without intellectual disabilities, on disease monitoring within and outside disease management programmes in 2015–2018^a^

**Characteristic**	**Enrolled in DMP, *n* (%)**		**Conditional OR (95% CI)**	**Not enrolled in DMP, *n* (%)**		**Conditional OR (95% CI)**
	
	**Patients with ID**	**Controls**		**Patients with ID**	**Controls**	
**CVD**	260	1316		277	1340	
Frequent consulters (≥9.1) per year^b^	130 (50.0)	371 (28.2)	**2.71^c^ (1.91 to 3.84)**	111 (40.1)	360 (26.9)	**1.78^c^ (1.28 to 2.49)**
Frequent CVD medication prescriptions (≥1.8) per year^d^	69 (26.5)	354 (26.9)	0.91 (0.62 to 1.32)	88 (31.8)	388 (29.0)	1.02 (0.73 to 1.43)
LDL measurement (only those aged <80 years) in 2018	230/250 (92.0)	1170/1268 (92.3)	0.80 (0.43 to 1.49)	81/269 (30.1)	531/1293 (41.1)	**0.60^c^ (0.42 to 0.87)**
Blood pressure test in 2018	205 (78.8)	1027 (78.0)	0.96 (0.65 to 1.42)	81 (29.2)	406 (30.3)	1.01 (0.72 to 1.41)

**Diabetes mellitus**	1105	4739		402	2302	
Frequent consulters (≥8.3) per year^b^	607 (54.9)	1582 (33.4)	**2.49^c^ (2.14 to 2.89)**	194 (48.3)	620 (26.9)	**2.83^c^ (2.05 to 3.89)**
Frequent diabetes mellitus medication prescriptions (≥1.8) per year^d^	348 (31.5)	1403 (29.6)	1.07 (0.91 to 1.25)	159 (39.6)	751 (32.6)	**1.46^c^ (1.10 to 1.95)**
HbA1c measurement in 2018	1000 (90.5)	4205 (88.7)	1.20 (0.94 to 1.53)	148 (36.8)	571 (24.8)	**1.85^c^ (1.34 to 2.55)**
Albuminuria test in 2018	856 (77.5)	3747 (79.1)	0.88 (0.74 to 1.05)	115 (28.6)	464 (20.2)	1.43 (0.99 to 2.07)

**COPD**	302	1437		393	2001	
Frequent consulters (≥9.3) per year^b^	174 (57.6)	490 (34.1)	**3.01^c^ (2.18 to 4.16)**	198 (50.4)	554 (27.7)	**2.88^c^ (2.22 to 3.73)**
Frequent COPD medication prescriptions (≥1.8) per year^d^	99 (32.8)	424 (29.5)	1.07 (0.78 to 1.46)	137 (34.9)	586 (29.3)	**1.28^c^ (0.99 to 1.66)**
At least two prescriptions of inhalation medication (R03A/B) in 2018 (only those with inhalation medication prescriptions)	110/139 (79.1)	519/655 (79.2)	1.04 (0.78 to 1.39)	80/99 (80.8)	282/406 (69.5)	**1.84^c^ (1.32 to 2.57)**
Smoking behaviour discussed in 2018	243 (80.5)	1123 (78.1)	1.32 (0.89 to 1.95)	61 (15.5)	268 (13.4)	1.36 (0.96 to 1.94)

a *The 2018 DMP guidelines state that all monitoring tests should take place once a year.*

b *Weighted average (by patients’ registration time in general practice) of number of contacts between patients and GPs (for example, regular consultations and home visits) between 2015 and 2018. For CVD, this average was 9.1 consultations per year, for diabetes mellitus 8.3 per year, and for COPD 9.3 per year.*

c P*<0.05.*

d *Weighted average (by patients’ registration time in general practice) of medication prescriptions between 2015 and 2018 across the study populations were 1.8 prescriptions per year for CVD, diabetes mellitus, and COPD. COPD = chronic obstructive pulmonary disease. CVD = cardiovascular disease. DMP = disease management programmes. ID = intellectual disabilities. LDL = low-density lipoprotein. OR = odds ratio.*

On average, between 2015 and 2018, patients with CVD, diabetes mellitus, and COPD received 1.8 prescriptions per year. Only among those not enrolled in DMPs were differences observed in frequency of medication prescriptions: patients with ID and diabetes mellitus (OR 1.46, 95% CI = 1.10 to 1.95) or patients with ID and COPD (OR = 1.28, 95% CI = 0.99 to 1.66) were more likely to receive ≥1.8 prescriptions per year than their controls (Table 2).

In those enrolled in CVD-DMP, there were no significant differences between patients with ID and their controls in the frequency of LDL checks (92.0% versus 92.3%) and blood pressure tests (78.8% versus 78.0%). In those not enrolled in CVD-DMP, patients with ID had a lower likelihood (30.1%) than their controls (41.1%; OR 0.60, 95% CI = 0.42 to 0.87) of receiving an LDL check (Table 2).

There were no significant differences between patients with diabetes mellitus and ID and their controls who were enrolled in the DMP in the frequency of HbA1c (90.5% versus 88.7%) and albuminuria measurements (77.5% versus 79.1%). HbA1c was more likely to be measured in patients with diabetes mellitus and ID who were not enrolled in the DMP (36.8%) than their controls (24.8%; OR 1.85, 95% CI = 1.34 to 2.55; Table 2).

In patients with COPD with and without ID who were enrolled in COPD-DMP, occurrence of receiving ≥2 prescriptions of inhalation medication (79.1% and 79.2%) and discussing smoking behaviour with care providers (80.5% and 78.1%) was similar. Patients with COPD and ID who were not enrolled in COPD-DMP were more likely to receive ≥2 prescriptions of inhalation medication (80.8%) than their controls (69.5%; OR 1.84, 95% CI = 1.32 to 2.57; Table 2).

## DISCUSSION

### Summary

This study compared enrolment in DMPs and disease monitoring for chronically ill patients with ID and their controls. Only patients with both diabetes mellitus and ID were more often enrolled in DMPs. Patients with ID enrolled in CVD-DMP, diabetes mellitus-DMP, or COPD-DMP received consultations more often than their enrolled controls. Among those not enrolled in a DMP, patients with ID received consultations, medication prescriptions, and routine examinations (apart from LDL measurement) more often than their controls. Despite the frequently reported care inequities between people with and without ID, the current study did not find evidence for limited access to DMPs for patients with an ID diagnosis and a chronic disease. Additional research is needed to explore whether chronic disease care meets the needs of this complex patient population.

### Strengths and limitations

This study is the first, to the authors’ knowledge, to examine DMP enrolment and disease monitoring for patients with ID and their controls by DMP enrolment using large-scale, individual-level data from a population-based primary care database. By linking datasets and using multiple methods to identify people with ID, the common problem of underreporting ID in medical records was reduced^34^ and therefore it has been possible to make more robust claims about a larger group of patients with ID than is possible by using solely GP records.^7^^,^^34^

Thanks to the matched study groups, the authors limited influences of sociodemographic factors and acknowledged the different demography of the ID population.^7^ As previous research has shown that these factors are important in the prevalence of chronic diseases and care utilisation in people with ID, as well as enrolment in DMPs,^15^^,^^35^^–^^39^ DMP enrolment and disease monitoring were explored in the current study without their interference.

Additionally, by utilising multiple methods to assess (non-)enrolment in DMPs, the risk of wrongly assigning patients was reduced and this provided a more precise image than by focusing solely on one method. Use of NPCD, containing information directly derived from clinical practice, allowed the examination of enrolment and disease monitoring as objectively as possible. However, the current findings do not fully comply with the 2018 benchmark from the national primary care organisation InEen. Unlike NPCD, this benchmark contains solely information from general practices, which are part of regional care groups.^40^ Future research should therefore consider regional differences.

Besides insufficient registration for DMP enrolment, registration for routine examinations and medication prescriptions is also most likely incomplete. Perhaps care providers registered routine examinations and medication prescriptions more thoroughly for those enrolled in DMPs because of DMP-related financial incentives.^4^ Although 85% of the total study population had no medication prescriptions, missing data occurred at a similar frequency for people with and without ID.

### Comparison with existing literature

To the best of the authors’ knowledge, DMP enrolment for patients with and without ID has not yet been studied, making comparison difficult. The current finding that patients with ID are often enrolled in diabetes mellitus-DMP could reflect their increased disease burden, as DMP enrolment is often higher among those with a higher disease burden and of younger ages.^33^^,^^35^^,^^41^ However, future research could examine the influence of socioeconomic determinants of enrolment in people with and without ID.

No studies, to the authors’ knowledge, have been identified that have examined disease monitoring by DMP enrolment for patients with ID. Disregarding DMP enrolment, similar to previous studies, the current study found that patients with ID have higher consultation rates than those without ID, and in general have high medication use.^8^^,^^36^^,^^42^^,^^43^ Although medication in people with ID may be prescribed more often to regulate challenging behaviour,^44^^,^^45^ this study was unable to consider medication type in the analyses.

The current finding that the frequency of routine examinations did not differ between those with and without ID conflicts with previous research. Most studies showed fewer tests in people with ID compared with the general population. Often, patients with both ID and CVD were found to receive fewer cholesterol and blood pressure tests, those with diabetes mellitus received microalbuminuria or HbA1c tests less often, and those with COPD discussed their smoking status or had smoking cessation advice less often than their controls.^19^^,^^20^^,^^22^^,^^24^ Several reasons can be proposed for these seemingly contradictory findings.

First, perhaps this study’s novel focus of investigating DMP enrolment before examining monitoring accounted for these differences: it could be that the financial incentives to stimulate DMP enrolment ensure increased compliance with chronic disease guidelines. Second, by innovatively linking administrative data to medical records, a more thorough identification of the people in the ID group was achieved. However, future research should further investigate these differences.

Among those not enrolled in DMPs, this study found that most tests for routine examinations were more often administered in patients with ID. Nevertheless, patients with (and without) ID not enrolled in DMPs still received fewer routine examinations than those enrolled. To the best of the authors’ knowledge, no previous studies have examined similar research questions. Research on patients’ experiences and outcomes is therefore required to interpret findings on routine examinations within and outside DMPs for patients with ID.

### Implications for practice

This study is the first, to the authors’ knowledge, to explore DMP enrolment and disease monitoring in the Netherlands for patients with chronic illness with and without ID. Although previous studies on disease monitoring among patients with ID often reveal a rather unfavourable image for these patients compared with the general population, this study shows that focusing on DMP enrolment can reveal other patterns: both patients with and without ID benefit hugely from DMP enrolment. As DMPs often improve clinical outcomes and quality of care for patients with chronic illness,^33^^,^^46^^,^^47^ policymakers should encourage healthcare insurers and care providers to provide suitable (financial) incentives for enrolling patients in DMPs.^35^ Patients with ID not enrolled in DMPs should be monitored regularly, as their already poorer health might further deteriorate without adequate recognition of health conditions.^48^ With their strong generalist skills, GPs have a solid foundation to address the complex healthcare needs of patients with ID.^48^ Despite the similarities in enrolment, awareness of patients’ individual care needs is thus an essential starting point for equitable and suitable chronic disease management.^15^^,^^49^

## Data availability

The data underlying this article were provided by Nivel by permission. Data will be shared on request to the corresponding author with Nivel’s permission.
